# Glycyrrhizic Acid Hydrogel Microparticles Encapsulated with Mesenchymal Stem Cell Exosomes for Wound Healing

**DOI:** 10.34133/research.0496

**Published:** 2024-10-14

**Authors:** Luting Zhang, Zhiqiang Luo, Hanxu Chen, Xiangyi Wu, Yuanjin Zhao

**Affiliations:** ^1^ Department of Rheumatology and Immunology, Nanjing Drum Tower Hospital, School of Pharmacy, Nanjing University of Chinese Medicine, Nanjing 210023, China.; ^2^ School of Biological Science and Medical Engineering, Southeast University, Nanjing 210096, China.; ^3^ Shenzhen Research Institute, Southeast University, Shenzhen 518071, China.; ^4^ Institute of Organoids on Chips Translational Research, Henan Academy of Sciences, Zhengzhou 450009, China.

## Abstract

Hydrogel microparticles have been proved to be curative to diabetic wounds. Current trends focus on the integration of bioactive matrix and their smart stimulus-responsive release to meet the complex demand of regeneration in diabetic wound. In this paper, we present novel stem cell exosome-encapsulated Chinese herb glycyrrhizic acid (GA) hydrogel microparticles for wound healing. The integrated GA endows the hydrogel microparticles with antibacterial properties, while the encapsulated exosomes impart them with pro-angiogenesis ability. In addition, as the black phosphorus is incorporated into these hybrid hydrogel microparticles, the release profile of GA and exosomes could be controllable under near-infrared irradiation due to the excellent photothermal effect of black phosphorus and the reversible phase transformation properties of GA. Based on these features, we have demonstrated that these microparticles can effectively kill bacteria, scavenge free radical, and promote angiogenesis from in vitro experiments. Besides, they could also markedly accelerate the wound healing process by down-regulating inflammation and promoting collagen deposition and angiogenesis in bacteria-infected in vivo diabetic wound. These results indicate that the proposed exosome-integrated GA hydrogel microparticles present great potential for clinical diabetic wound treatment.

## Introduction

Chronic wounds caused by diabetes frequently occur [1–3]. They are susceptible to bacterial infections, which are accompanied with persistent inflammation at the infectious site and delayed wound healing [4–6]. Once the diabetic wound is not treated timely, it may be preceded with serious lesions such as amputation and sepsis [7,8]. In the clinic, treatments of infected diabetic wounds are normally initiated by the utilization of antibacterial drugs including antibiotics and inorganic silver nanoparticles to prevent early bacterial infection, followed by the application of vascular endothelial growth factor for regenerative therapeutics [9]. Although with many successes, the clinical values of these therapies are usually hampered by the antibiotic resistance, biotoxicity of inorganic nanoparticles, short half-time of bioactive molecular, and high cost [10,11]. As an alternative, various biomedical hydrogel microparticles have been fabricated to administrate the therapeutics, prolonging drug duration and reducing their toxic side effects [12–20]. Especially, their release of encapsulated bioactives could be controlled by external stimuli when their components are incorporated with some 2-dimensional additives like graphene oxide and black phosphorus (BP) [21–24]. However, most of these responsive hydrogel microparticles still have limitations in terms of biocompatibility, multiple biological effects, tailored release profile, and ease of manufacture. Therefore, new responsive hydrogel microparticles with natural components and bioactive drugs are highly anticipated.

**Fig. 1.**
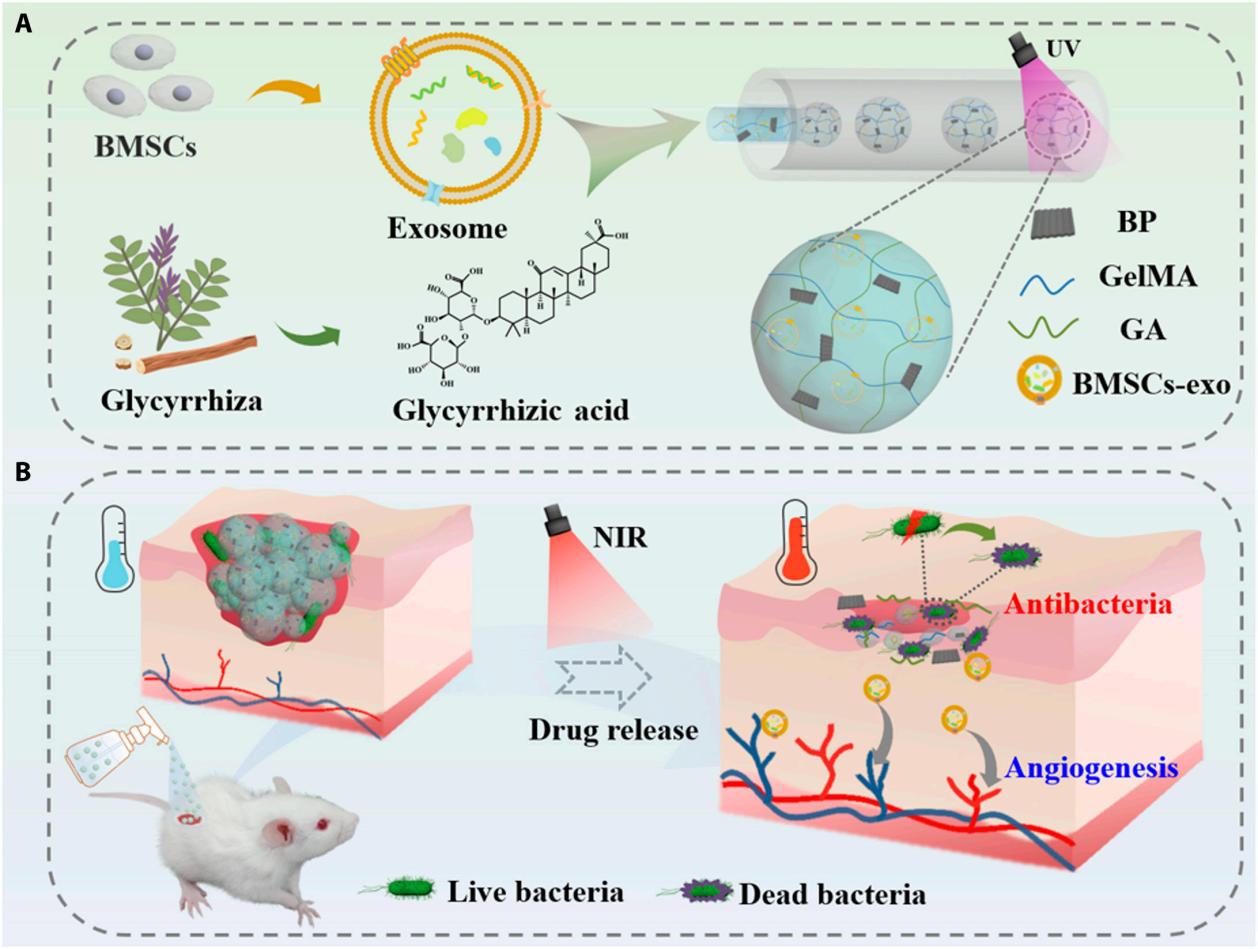
(A) Schematic diagram of microfluidic preparation of exosome-encapsulated Chinese herb hydrogel microparticles. (B) Healing process of the diabetic wound accelerated by hydrogel microparticles with exosomes and Chinese herb.

Here, we proposed novel glycyrrhizic acid (GA) hydrogel microparticles encapsulated with stem cell-derived exosomes for wound healing, as shown in Fig. 1. GA is an active ingredient extracted from natural glycyrrhiza [25]. It has been reported with the biological properties of antibacterial, anti-inflammation, antioxidant, biocompatibility, and biodegradability, which are all beneficial in promoting wound healing [26–29]. In addition, GA has the advantages of low cost, easy accessibility, and high stability. In contrast, exosomes are nanoscale vesicles secreted by cells, which carry many endogenous cytokines and chemokines [30–32]. Especially, the exosomes derived from bone mesenchymal stem cells (BMSCs) are found with the high levels of various wound healing-promoting growth factors like interleukin-6 (IL-6), IL-8, and CXCL-1 [33,34]. They have performed the properties to reduce wound inflammation and promote re-epithelialization and angiogenesis [35]. Interestingly, the microfluidic emulsification system allows precise control of droplet size, facilitating high throughput and reducing sample volume. Thus, the integration of GA and BMSC-derived exosomes into hydrogel microparticles with stimulus responsiveness by the microfluidic emulsification system was expected to harvest the promising patches with multifunction for wound healing.

In this paper, we employed a microfluidic emulsification system to fabricate the exosomes and Chinese herb GA-incorporated BP hydrogel microparticles for the treatment of diabetic wounds. The precursor for the microfluidic emulsification was composed of GA extracted from natural glycyrrhiza, exosomes derived from BMSCs, and BP tagged gelatin methacryloyl (GelMA) solution. The resultant hydrogel microparticles were endowed with the antibacterial, anti-inflammatory, and pro-angiogenic capacities. Besides, the excellent photothermal conversion performance of BP under near-infrared (NIR) irradiation contributed to the increase of the local temperature of microparticles. The generated heat could induce the transformation of GA from gel to sol due to the reversible phase transition property of GA, thus accelerating the release of encapsulated exosomes and GA. Cell experiments and antibacterial tests confirmed that the GA microparticles encapsulated with BMSC exosomes have excellent antibacterial and angiogenic ability. In addition, these exosome-integrated GA microparticles could also accelerate the healing process of bacteria-infected diabetic wound by promoting vascularization and reducing the level of inflammatory response. Thus, we believed that our Chinese GA herb hydrogel microparticles encapsulated with bioactive exosomes have presented great potential for clinical application.

## Results and Discussion

**Fig. 2.**
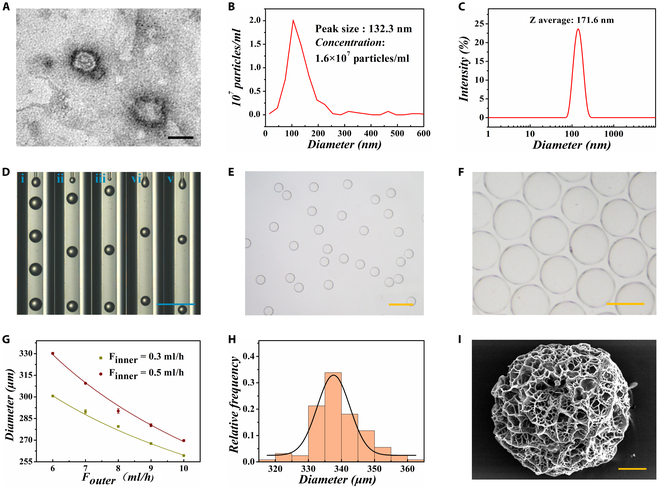
Preparation and characterization of BMSCs-exo-encapsulated GA hydrogel microparticles. (A) TEM image of BMSCs-exo. (B and C) Concentration and size distribution of BMSCs-exo. (D) Real-time generation of droplets in the microfluidic channel at the outer phase flow rate of (i) 1 ml/h, (ii) 2 ml/h, (iii) 3 ml/h, (iv) 4 ml/h, and (v) 5 ml/h. Inner phase flow rate: 0.3 ml/h. (E) Optical image of spherical droplets. Scale bar, 600 μm. (F) Optical image of photopolymerized microparticles. (G) Relationship of droplet size with the change of flow rate. (H) Histogram of size distribution of microparticles. (I) SEM image of the microparticles. Scale bars, 100 nm (A), 1 mm (D), 600 μm (E), 300 μm (F), and 50 μm (I).

In this study, GelMA was self-synthesized by reacting gelatin with methacrylic anhydride (MA). As shown in Fig. S1, GA molecules were integrated with GelMA hydrogel, and the resultant hybrid hydrogel could undergo a reversible gel–sol phase transition with an increase in temperature from 37 to 50 °C. Such property facilitated the construction of the smart thermal-responsive delivery system. Subsequently, the exosomes were isolated from the supernatant culture medium of BMSCs through ultracentrifugation at 44,000 rpm. These BMSC-secreted exosomes (BMSCs-exo) were featured with double-layered membranes resembling cups, as characterized by the transmission electron microscopy (TEM) in Fig. 2A. Meanwhile, nanoparticle tracking analysis revealed that the maximum concentration of BMSCs-exo was approximately 1.435 × 10^7^ particles/ml with a homogeneous diameter of 105 nm (Fig. 2B). In addition, as shown in Fig. 2C and Fig. S2, the dynamic light scattering (DLS) results demonstrated that temperature and ultraviolet (UV) irradiation had a negligible influence on the hydrodynamic size of BMSCs-exo.

The fabrication of BMSCs-exo-encapsulated herb GA hydrogel microparticles was achieved by a self-prepared co-flow microfluidic device. To be specific, the GA and BMSCs-exo were mixed with GelMA and BP to form the inner phase, while soybean oil acted as the outer phase. Under the action of interfacial tension and shear force, inner phase solution was sheared into monodisperse droplets. The droplet diameter could be adjusted through regulation of the 2-phase flow rate. When the flow rate of the inner phase remained constant, the droplet diameter showed a negative correlation with the flow rate of the outer phase (Fig. 2D). Statistical results of droplet diameter change are shown in Fig. 2G. Under UV irradiation, the droplets were polymerized into hydrogel microparticles due to the crosslinking of GelMA. The optical images of microdroplets and polymerized microparticles are shown in Fig. 2E and F. These microparticles had good monodispersity at an average size of 339 μm (Fig. 2H). The microstructure of freeze-dried microparticles was porous, as characterized by scanning electron microscopy (SEM) (Fig. 2I). To better investigate the distribution of exosomes in microparticles, the BMSCs-exo was stained by calcein-AM. The fluorescent image demonstrated the successful encapsulation and uniform spatial distribution of BMSCs-exo (Fig. S3).

**Fig. 3.**
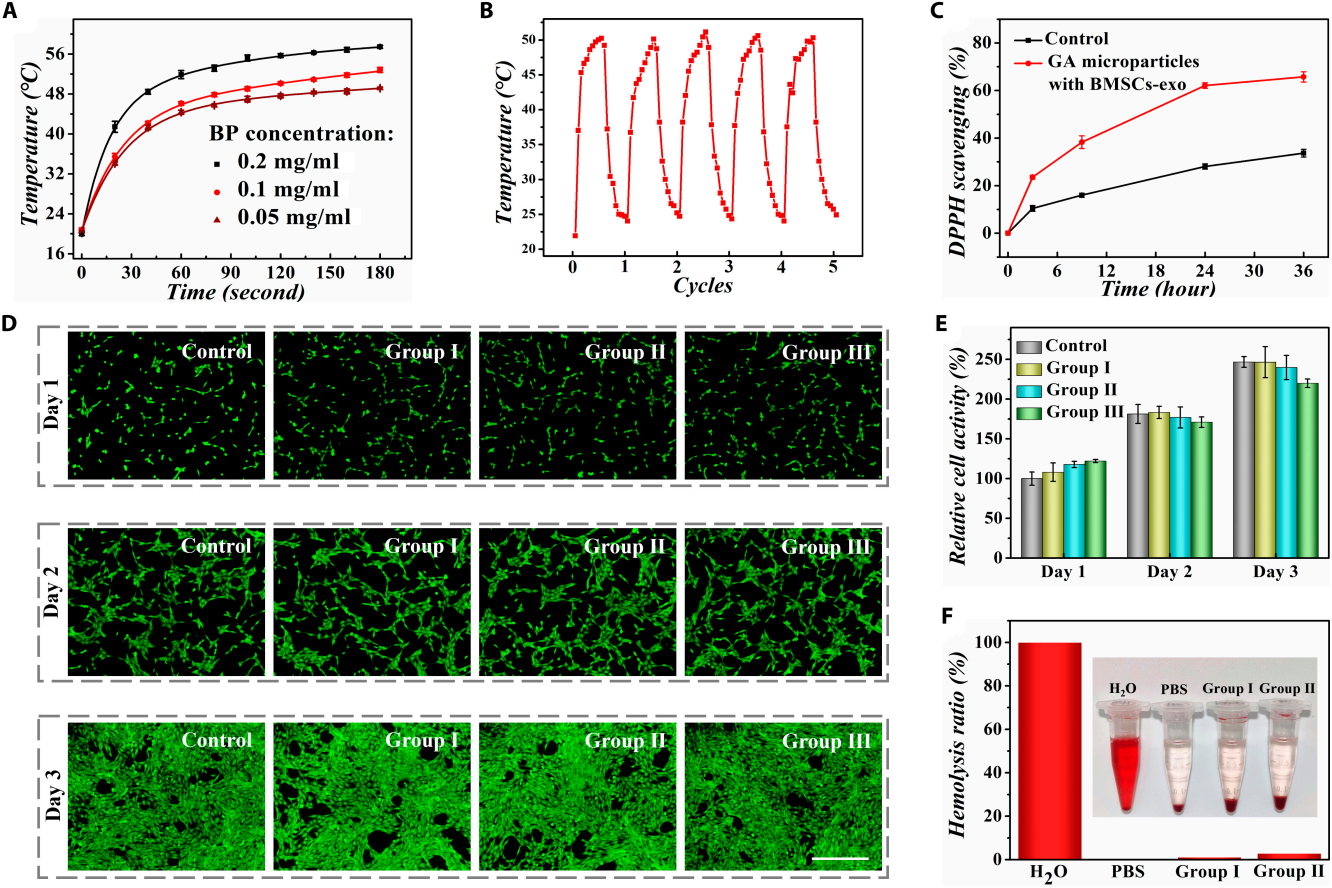
Multifunction evaluation of GA microparticles encapsulated with BMSCs-exo. (A) Plot of the temperature variations of microparticles containing different concentrations of BP under NIR irradiation. (B) Plot of the temperature variations of microparticles during 5 cycles. (C) Characterization of DPPH clearance activity of GA microparticles containing BMSCs-exo. (D) Fluorescent images of cells treated with different microparticles during 3 d. (E) Quantified viability of cells of different groups. *n* = 3 for each group. (F) Hemolysis assessment of microparticles. Scale bar, 500 μm (D).

Benefiting from the integration of several functional components, the resultant hydrogel microparticles were imparted with multifunction of photothermal responsiveness, antioxidation, high biocompatibility, antibacterial, and promotion of cell migration, which were proved in the following in vitro tests. The integration of BP into hydrogel microparticles endowed them the capability to produce heat when exposed to NIR irradiation. As shown in Fig. 3A, under the irradiation of NIR laser with a power of 2.69 W/cm^2^, within 3 min, the local temperatures of microparticles with BP concentrations of 0.05, 0.1, and 0.2 mg/ml rose to 49.1, 52.8, and 57.5 °C, respectively. Based on the transformation of GA from the gel state to the sol state at 50 °C, the BP concentration was set as 0.1 mg/ml to induce the phase transition of GA for acceleration of drug release. The cyclic test containing 5 consecutive on/off cycles of the NIR laser was subsequently carried out to assess the photothermal conversion stability of microparticles. In each cycle, the microparticles were rapidly heated to 50 °C when the NIR was switched on and promptly cooled back to the initial temperature once the NIR was switched off (Fig. 3B). These results indicated that the microparticles possessed a stable NIR-responsive heating capability.

With NIR stimulation, the drug release from GA microparticles can be controlled. NIR irradiation obviously accelerated the drug release from GA microparticles compared with no NIR irradiation, as shown in Fig. S4. Then, we verified the reactive oxygen species (ROS)-scavenging capacity of GA microparticles containing BMSCs-exo. 1′-Diphenyl-2-picrylhydrazyl radical (DPPH) was used as the model of free radical. It was observed that the content of DPPH dramatically decreased after the treatment of microparticles, showing their excellent antioxidant performance (Fig. 3C). The excellent antioxidant activities of microparticles would be beneficial to scavenge the harmful ROS in wounds to accelerate their healing process.

To validate the biocompatibility of the fabricated hydrogel microparticles, NIH 3T3 cells were selected to be incubated with blank hydrogel microparticles without GA and BMSCs-exo (group I), GA microparticles containing BMSCs-exo (group II), and GA microparticles containing BMSCs-exo under NIR irradiation (group III). Cells cultured with pure culture medium were set as the control group. The fluorescent images of cells stained by calcein-AM showed that the cells in each group showed a healthy morphology during 3 d of culture, as shown in Fig. 3D. Furthermore, 3-(4,5-dimethylthizaol-2-yl)-2,5-diphenylterazolium (MTT) assay was conducted to assess biocompatibility. Results were consistent with the data of fluorescent staining, demonstrating that the material had satisfactory biocompatibility (Fig. 3E). Besides, a hemolysis test between hydrogel microparticles and erythrocytes was also carried out to verify the blood compatibility of hydrogel microparticles and encapsulated drugs. The hemolysis rates of group I and group II were 1.1% and 2.9%, respectively. Both of them were less than 5%, indicating the good biocompatibility of these microparticles (Fig. 3F).

**Fig. 4.**
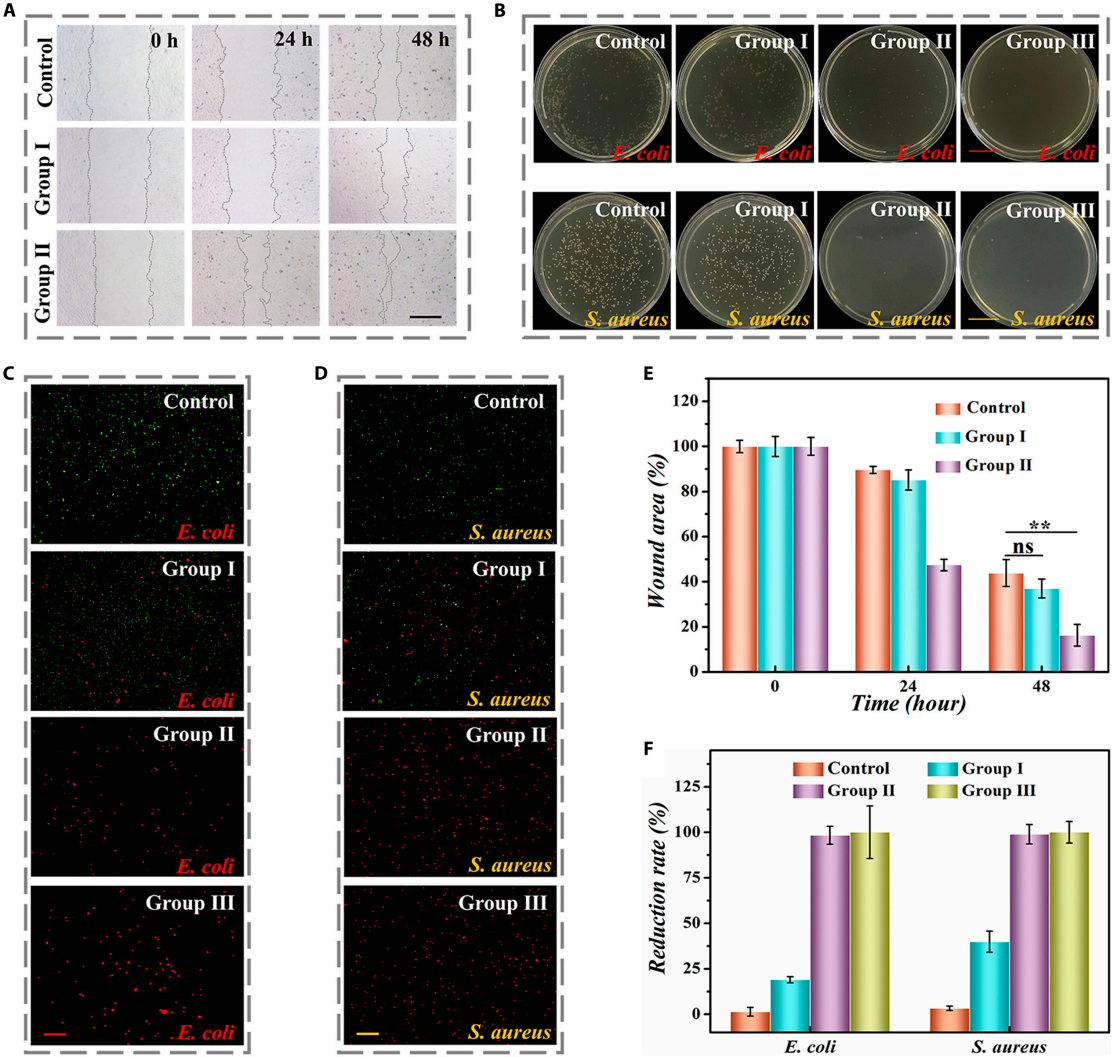
Characterization of scratch assay and in vitro antibacterial experiment. (A) Representative images of the scratch wound. (B) Images of bacterial colonies on culture plates of different groups. (C and D) Fluorescent images of *E. coli* and *S. aureus* stained by SYTO (green) and propidium iodide (PI) (red). (E) Quantification of scratch test (*n* = 3). (F) Reduction rates of *E. coli* and *S. aureus* in different groups. Scale bars, 400 μm (A), 2 cm (B), and 50 μm (C). **P < 0.01; ns, not significant.

Angiogenesis can provide oxygen and nutrients to the wound, contributing to the repair and regeneration of wound tissue. We performed in vitro angiogenesis experiments to investigate the promotion of BMSCs-exo released from microparticles to angiogenesis. It was found that microparticles without BMSCs-exo could not promote angiogenesis, while the microparticles with BMSCs-exo greatly improved the vascular cell cyclization (Fig. S5). Considering that cell migration was involved in wound healing, we examined the impact of GA microparticles with BMSCs-exo on cell migration through the scratch assay. The presence of BMSCs-exo obviously facilitated the closure of scratches (Fig. 4A), and the closure rate of group II was close to 90% compared to the control group (55%), demonstrating good promotion ability for wound healing (Fig. 4E).

It is worth mentioning that killing pathogenic bacteria can efficiently prevent wound infection, inhibit inflammation, relieve pain, and promote wound healing. The antimicrobial properties of GA microparticles with BMSCs-exo were investigated by coincubation with *Escherichia coli* and *Staphylococcus aureus*. In preliminary test, we observed that 15% w/v GA could achieve a killing ratio of nearly 100% (Fig. S6). Therefore, the concentration of GA in the microparticles was set as 15% w/v for subsequent experiments. Then, the antimicrobial property of microparticles was studied, where 4 groups were established, including the control group, blank hydrogel microparticles (group I), GA microparticles with BMSCs-exo (group II), and GA microparticles with BMSCs-exo under NIR irradiation (group III). It was found that group III appeared the best antibacterial activity, whose killing rate of *E. coli* and *S. aureus* reached nearly 100%, as shown in Fig. 4C, D, and F. In addition, the agar plate dilution test was performed, where nearly no colony was observed in group II and group III in contrast to the control group. These results further confirmed the excellent antimicrobial capacities of GA microparticles with BMSCs-exo (Fig. 4B).

To explore the practical value of GA microparticles with BMSCs-exo for wound healing, we established a model of *S. aureus*-infected diabetic wounds on rats. The successful modeling of diabetics was realized by periodic injection of streptozotocin, which was proved by the detected high blood glucose levels in rats (Fig. S7). The diabetic rats were freely grouped and received treatment of phosphate-buffered saline (PBS) (Control), blank microparticles (BM), drugs of GA and BMSCs-exo (Drug), GA microparticles containing BMSCs-exo (BM-Drug), and GA microparticles with BMSCs-exo together with regular NIR radiation (BM-Drug-NIR). At day 0, the NIR-triggered heating behavior of the microparticles was first verified in vivo on the dorsal skin of rats. Thermal images revealed that the temperature of the wound area covered by microparticles increased from 35.1 to 52.8 °C within 3 min, while that of the surrounding tissues showed little changes (Fig. S8). It was indicated that these microparticles possessed desirable photothermal conversion properties in vivo as well.

**Fig. 5.**
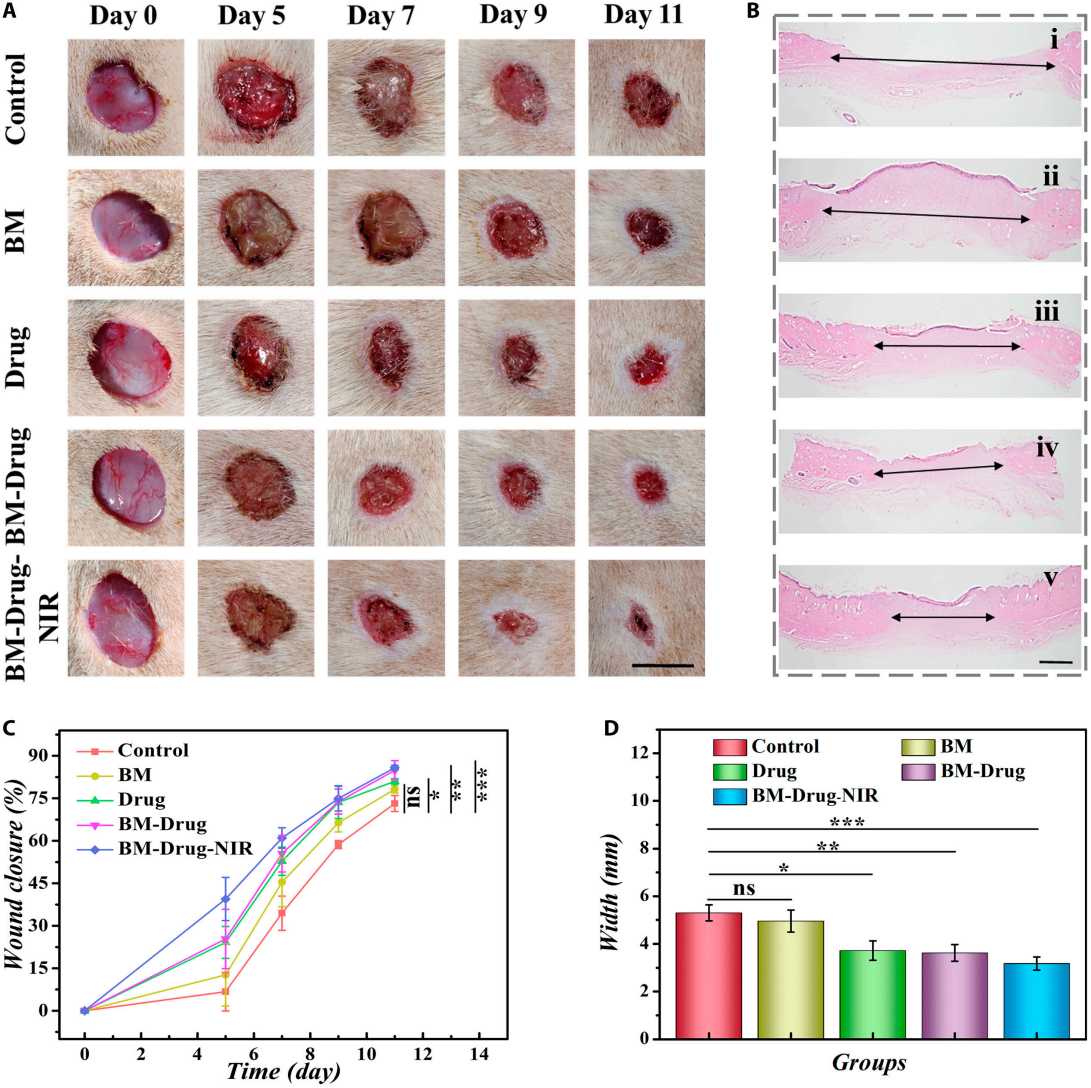
Effects of different treatments on the infected diabetic wounds. (A) Representative images of the wounds in different groups. (B) H&E staining images of skin tissues. i, ii, iii, iv, and v in (B) represent control group, BM group, Drug group, BM-Drug group, and BM-Drug-NIR group,respectively. (C) Data analysis of wound closure in different groups. (D) Quantitative analysis of the granulation tissue width on day 11. Scale bars, 1 cm (A) and 1 mm (C). *P < 0.05, **P < 0.01, ***P < 0.001.

The wound healing process was photographed on days 0, 5, 7, 9, and 11 after treatments. Then, the wound area at different time points was calculated for quantitative analysis (Fig. 5A and Fig. S9). On day 2, the status of bacterial infection on the diabetic wounds was examined by the agar plate method. It was found that compared with PBS treatment, the experimental group after NIR irradiation formed fewer colonies (Fig. S10). At day 11, the wound healing condition of the control group was the worst, and the healing rate of the Drug and BM-Drug groups was higher than that of the BM group. The BM-Drug-NIR group showed that the highest wound closure rate of up to 87% for the released GA and BMSCs-exo from microparticles could effectively promote wound healing (Fig. 5C). To further explore the status of the wound regeneration, the regenerated area of each group at day 11 was stained by hematoxylin-eosin (H&E) to assess the width of regenerated tissues (Fig. 5B). Compared to the control group, the BM-Drug-NIR group showed a smaller granulation tissue width, supporting better wound healing as well (Fig. 5D).

**Fig. 6.**
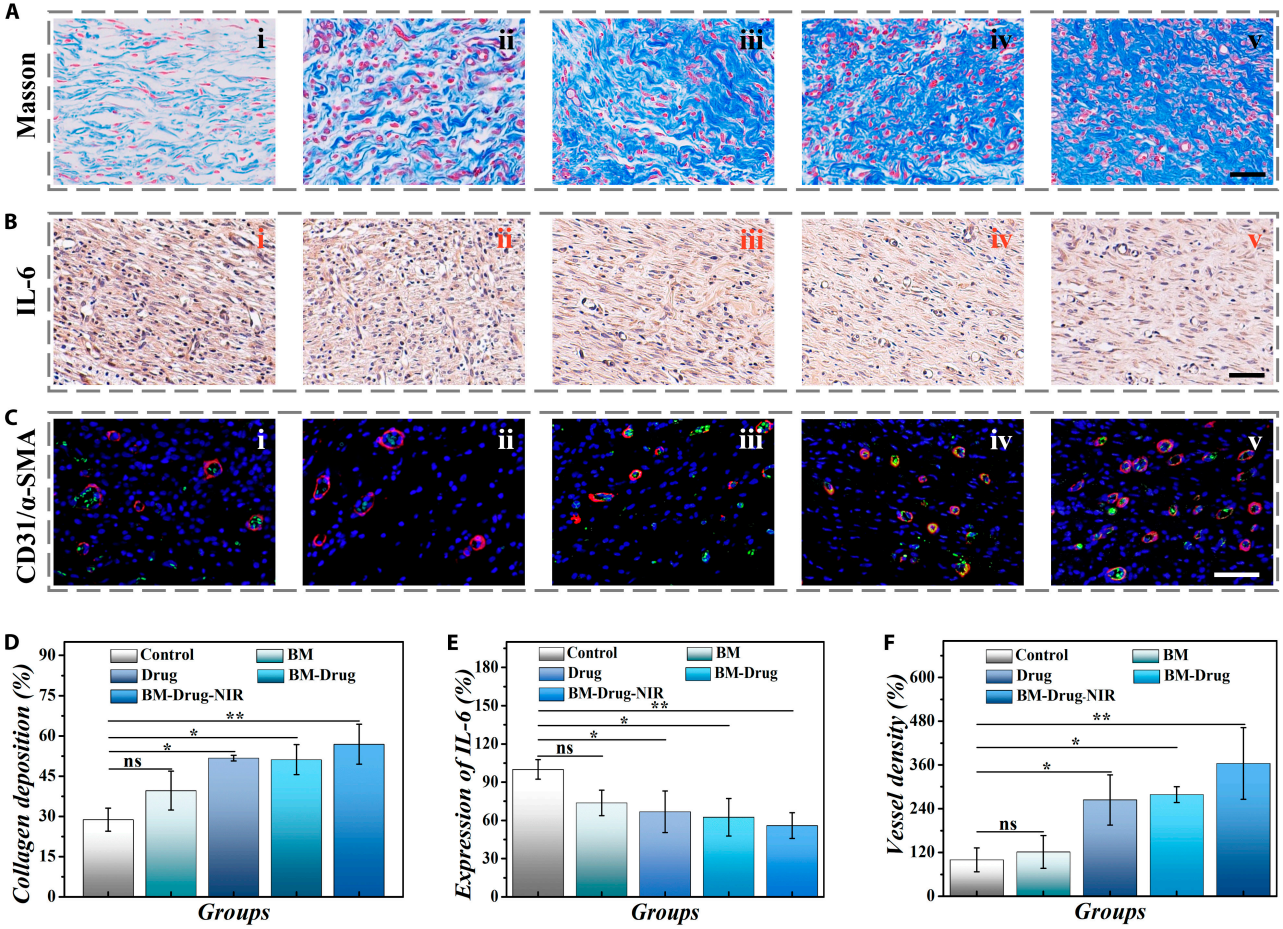
Study of biological mechanisms of GA microparticles with BMSCs-exo to promote wound healing. (A) Masson staining of the regenerated tissues in different groups at day 11. (B) IL-6 immunohistochemistry staining images of the wounds in 5 groups at day 11. (C) Double immunofluorescent staining of CD31 (green) and α-SMA (red) at day 11. i, ii, iii, iv, and v in (A) to (C) represent control group, BM group, Drug group, BM-Drug group, and BM-Drug-NIR group,respectively. (D) Analysis of collagen deposition of different groups. (E) Analysis of relative expression of IL-6 of different groups. (F) Analysis of CD31/α-SMA of different groups. Scale bars, 50 μm (A to C). *P < 0.05, **P < 0.01.

To further investigate the remodeling level of the wound, histochemical staining of regenerated tissues was conducted. As evidence for cell growth, collagen has been proven to be able to enhance cell proliferation/differentiation and create a more favorable microenvironment. To reveal the state of collagen deposition, Masson staining was carried out. It was found that the control group expressed the lowest collagen. In contrast, the BM-Drug-NIR group showed denser collagen deposition along with aligned collagen fibers (Fig. 6A). The expression of inflammatory factors reflects the level of inflammation at the wound site. IL-6 staining results showed that the BM-Drug-NIR group had the least inflammation, followed by the BM-Drug and Drug groups, and the most severe inflammation was observed in the control group (Fig. 6B). Additionally, the level of angiogenesis, another key indicator for evaluation of the healing process, was assessed. The results of double immunofluorescence staining for CD31 and α-smooth muscle actin (α-SMA) are shown in Fig. 6C. The control and BM groups had fewer vascular structures, while the other 3 groups benefited from BMSCs-exo to promote angiogenesis. Specifically, the BM-Drug and BM-Drug-NIR groups achieved continuous drug release at the wound site, extending the release time and resulting in a denser vascular distribution. Notably, the BM-Drug-NIR group achieved intelligent drug release, resulting in the densest vascular distribution. The statistical data of these biomarkers were consistent with the results of the staining images (Fig. 6D to F). These findings showed the improvement of tissue reconstruction and wound healing process in the BM-Drug-NIR group, revealing the great curative effect of GA microparticles with BMSCs-exo.

## Conclusion

In conclusion, we prepared multifunctional herbal hydrogel microparticles encapsulated with BMSCs-exo. These hydrogel microparticles with photothermal responsiveness were fabricated by microfluidics technology, consisting of GelMA, BP, herbal GA, and encapsulated BMSCs-exo. The resultant hydrogel microparticles could maintain the bioactivity of the BMSCs-exo, along with sustainable release at the wound site. In addition, the integration of small-molecule GA imparted the resultant hydrogel microparticles with good antibacterial properties for infection prevention. For the treatment of diabetic wounds, the multifunctional herbal hydrogel microparticles effectively promoted the formation of granulation tissues with enhanced collagen deposition, induced angiogenesis, and inhibited the inflammatory response for wound healing. Therefore, it is believed that such multifunctional microparticles could find practical applications in delivering bioactive molecules for the treatment of diabetic wounds.

## Materials and Methods

### Materials

GA was purchased from Shanghai Yuanye Biologicals. Gelatin, MA, and dimethyl sulfoxide (DMSO) were obtained from Sigma-Aldrich. GelMA was self-prepared by the reaction of gelatin and MA. MTT reagent was purchased from J&K Scientific Ltd., Shanghai. 2-Hydroxy-2-methylpropiophenone (HMPP) and rhodamine B were provided by Aladdin. BP was purchased from Centrum Nano. The components of cell complete culture medium were provided by Gibco. Calcein-AM was acquired from Thermo Fisher Scientific, USA. SYTO was purchased from KeyGEN BioTECH. Propidium iodide (PI) was acquired from Beyotime. DPPH was purchased from Acmec biochemical. All antibodies used for tissue staining were provided by Servicebio. All reagents were used as received. The Cell Bank of the Chinese Academy of Sciences (China) provided human umbilical cord endothelial cells (HUVECs), BMSCs, and NIH 3T3 cells. Besides, the male Sprague–Dawley (SD) rats (8 to 12 weeks) were obtained from Drum Tower Hospital, and the Animal Investigation Ethics Committee of Nanjing Drum Tower Hospital permitted the animal experimental programs.

### Preparation of GelMA

Gelatin (10 g) was dissolved in 100 ml of PBS, stirred, and heated in the water bath at 60 °C for 1 h. Subsequently, 10 ml of MA was added dropwise to the gelatin solution. Once the solution was milky white, 400 ml of PBS was added and stirred for 2 h. When the solution became clear and pale yellow, it was poured into a dialysis bag and dialyzed in deionized water at 45 °C for 7 d. The final dialyzed solution was freeze-dried and stored at −20 °C.

### BMSC culture and BMSCs-exo collection

BMSCs were cultured in Dulbecco’s modified Eagle’s medium/F12 complete medium. The cell culture media were collected after filtration and centrifuged in an ultracentrifuge (Beckman Coulter, Optima) at 44,000 rpm for 1.5 h, and then the harvested sediments were resuspended with PBS and centrifuged again for 1 h. The collected precipitate was resuspended in PBS and then filtered through a sterile filter (0.22 μm, Millipore) to obtain BMSCs-exo suspension.

### Construction of single-emulsion microfluidic device

The microfluidic device comprised an inner-phase round capillary, an outer-phase round capillary, and a square glass capillary for observation. The outer-phase capillary possessed an inner and outer diameter of 580 μm and 1 mm, respectively, which was hydrophobized before capillary assembly. The inner capillary was pulled into a cone by a tube puller and then sandpapered until its inner diameter reached 100 μm. The square observation capillary was at an inner size of 1.05 mm. To assemble the chip, the square capillary was fixed in the middle of the slide by glue. The outer and inner phase capillaries were fixed through the square capillary so that the nozzle of the inner phase capillary was centered on the outer phase capillary. The joints between the tubes were sealed with a dotting needle and glue.

### Preparation of GA hydrogel microparticles encapsulating BMSCs-exo

The pregel solution consisted of 10 w/v % GelMA, 1 × 10^8^ particles/ml BMSCs-exo, 15% GA, 1% HMPP, and BP. Then, a microfluidic platform was established with pregel solution and soybean oil as inner and outer phases, respectively. A hot air blower was placeded next to the platform to provide a warm environment. The 2 phases were put into syringes and connected to the microfluidic chip through a sampling needle and a polyethylene hose. The flow rates were adjusted by micro-injection pumps. A heating plate was placed between the syringe and the microinjection pump to control the temperature. The inner phase solution was sheared by the soybean oil in the outer phase capillary to form monodispersed droplets, which were collected in the soybean oil. The microparticles were obtained after photopolymerization of droplets under irradiation of UV light (365 nm, 100 W) for 1 min.

### Characterization of BMSCs-exo and hydrogel microparticles

Microstructural characterization of GA microparticles with BMSCs-exo was obtained with a field emission scanning electron microscope (Nikon A1). The morphology of BMSCs-exo was gained by a transmission electron microscope (JEOL, JEM-2100). Optical images were required by a stereoscopic microscope (JSZ6S, Jiangnan novel optics) equipped with a charge-coupled device camera (Olympus, DP30BW). The BMSCs-exo concentration was measured by Nanosight (ZetaView, Particle Metrix). Fluorescence images were accessed by a fluorescence microscope (Olympus, CKX41). DLS particle sizer (Malven Zetasizer NanoZS, Malvern) was used to characterize the size distribution of BMSCs-exo.

### Photothermal performance

The hydrogel microparticles were irradiated with a NIR laser (2.69 W/cm^2^) (BP concentration: 0.05, 0.1, and 0.2 mg/ml). The temperature changes were recorded by a thermal imager. For the on/off cycles, the NIR irradiation lasted for 3 min, and the next cycle began after cooling for 3 min.

### In vitro drug release experiment

We used rhodamine B to simulate the drug release from GA microparticles. The initial concentration of rhodamine B was 2 mg/ml. GA microparticles encapsulated with rhodamine B were soaked in 1 ml of PBS. PBS (100 μl) was periodically sucked, and then the absorbance (Abs) was detected at the wavelength of 570 nm. After sampling, GA microparticles were irradiated with NIR (2.69 W/cm^2^) for 3 min, and new PBS was supplemented in time.

### Antioxidant test

DPPH was dissolved in ethanol solution to achieve an Abs value of nearly 1.0. The control group was 1 ml of DPPH, while the experimental group was additionally added with GA microparticles with BMSCs-exo. Subsequently, the samples were incubated at 37 °C on a shaker, and the Abs at 517 nm was determined at 0, 3, 9, 24, and 36 h. The formula for calculating the antioxidant activity was as follows:

$$\text{Antioxidant activity}\;\left(\%\right)=\frac{{\text{Abs}}_{\text{control}}-{\text{Abs}}_{\text{sample}}}{{\text{Abs}}_{\text{control}}}$$

where Abs_control_ represents the Abs detected at the initial moment and Abs_sample_ represents the Abs of the sample at the corresponding moment.

### In vitro biocompatibility experiment

The experiment included 4 groups with 3 parallel samples in each group. The control group was cultured by pure culture medium, and the experimental groups included blank microparticles without GA and BMSCs-exo (group I), GA microparticles with BMSCs-exo (group II), and GA microparticles with BMSCs-exo together with treatment of NIR irradiation (group III). NIH 3T3 cells in 4 groups were incubated in the wells of a 48-well plate for 1, 2, and 3 d, respectively. When the cultivation was finished, the solution was sucked out and the medium containing MTT was added. After 4 h, the medium was sucked out and 500 μl of DMSO was added to dissolve the formazan crystal. Finally, cell viability was calculated by measuring the corresponding Abs. In addition, the cell status was observed daily after calcein-AM staining.

### Hemolysis test

Fresh SD rat erythrocyte suspension with a volume ratio of 2% was prepared. Four centrifuge tubes were added with 1 ml of fresh erythrocyte suspension. In addition, 1 ml of distillation-distillation water (ddH_2_O) and 1 ml of PBS were added to the positive control group and negative control group, respectively. Furthermore, blank hydrogel microparticles without GA and BMSCs-exo (group I), and GA microparticles with BMSCs-exo (group II) were added to the experimental groups. After standing at 4 °C overnight, the hemolysis of the 4 groups was observed. Subsequently, Abs_545_ of the supernatant was detected and the hemolysis rate was estimated according to the following equation:

$$\text{Hemolysis ratio}=\frac{{\text{Abs}}_{\text{sample}}-{\text{Abs}}_{\text{PBS}}}{{\mathrm{Abs}}_{\text{dd}H_{2}O}-{\text{Abs}}_{\text{PBS}}}$$

where Abs_sample_ represents the Abs of the experimental group and Abs_ddH2O_ and Abs_PBS_ represent the Abs of the positive control and negative control, respectively.

### Tube formation experiment

The bottom of the 48-well plate was painted with magerial and then gelled at 37 °C. HUVECs (3 × 10^4^) were seeded in the wells, which were treated with blank medium (control) and GA microparticles without (group I) and with (group II) BMSCs-exo. After 4 h, HUVECs were stained with calcein-AM for observation. Tube length was measured by ImageJ software.

### Scratch assay

Six-well plates were seeded with 6 × 10^5^ HUVECs per well, and cells were cultured until they formed a complete monolayer in the wells. The cellular monolayer was gently scraped with the tip of a pipette, and then the exfoliated cells were washed with PBS. The control group remained untreated, while the other 2 groups added GA microparticles without (group I) and with (group II) BMSCs-exo. Each group had 3 parallel experiments. The samples were photographed at 0, 24, and 48 h.

### Antibacterial test

We selected *E. coli* and *S. aureus* to assess the antibacterial efficacy of the materials. The bacteria were diluted to the turbidity of 0.5 McFarland’s standard with PBS. GA solutions with concentrations of 5%, 10%, and 15% were cocultured with the bacteria at 37 °C for 24 h, and then the optimal antibacterial concentration was obtained by live/dead staining results. Subsequently, the bacterial suspensions were coincubated with fresh PBS (control group), blank hydrogel microparticles without GA and BMSCs-exo (group I), GA microparticles with BMSCs-exo (group II), and GA microparticles with BMSCs-exo under NIR irradiation (group III) for 24 h at 37 °C. After that, these bacteria were stained by SYTO and PI for live/dead staining, followed by observation under fluorescence microscopy. In addition, bacterial suspensions that had been cocultured for 12 h were diluted, spread on agar plates, and placed in incubation at 37 °C overnight.

### Wound healing study

Firstly, a diabetic wound model with *S. aureus* infection was established. SD rats were freely allocated into 5 groups. Rats were intraperitoneally injected with 1% w/v streptozotocin solution at 70 mg/kg. The blood glucose levels of all rats were measured by a glucometer. After 7 d, these SD rats were anaesthetized and circular wounds were established on their back, where 100 μl of *S. aureus* suspension was added on the surface. Control rats received PBS to flush the wounds. The remaining experimental groups received treatment with blank microparticles without GA and BMSCs-exo (BM), GA and BMSCs-exo (Drug), GA microparticles with BMSCs-exo (BM-Drug), and GA microparticles with BMSCs-exo and regularly irradiated with NIR (BM-Drug-NIR). The wounds were photographed on days 0, 5, 7, 9, and 11 after treatment. On day 2, the wounds were washed with 100 μl of PBS, and the collected washings were used for agar plate smears. At day 11, the rats were killed, and all wound tissues were cut and soaked in paraformaldehyde. Finally, the embedded sections of wound tissues were used for immunohistochemical and histological analyses.

### Statistics analysis

Each test was conducted with a minimum of 3 repetitions. Image processing utilized ImageJ, while statistical analysis was performed using Origin Lab software. Results were expressed as mean ± standard deviation. Statistical analysis was carried out using one-way analysis of variance (ANOVA) to determine the degree of significance by the software of SPSS. Statistical significance was defined as ns: no significant, 0.01 < *P < 0.05, **P < 0.01, ***P < 0.001.

## Data Availability

Data will be made available on request.
