# Supplementary material for: Glycyrrhizic Acid Hydrogel Microparticles Encapsulated with Mesenchymal Stem Cell Exosomes for Wound Healing
Source: Research (Wash D C). 2024 Oct 14;7:0496. doi: 10.34133/research.0496 (PMC11471873; doi:10.34133/research.0496)
Supplement: Supplementary 1 — Figs. S1 to S10 [file research.0496.f1.docx]

**Supplementary Materials**

**Glycyrrhizic Acid Hydrogel Microparticles Encapsulated with MSC Exosomes for Wound Healing**

**Luting Zhang^1^, Zhiqiang Luo^2^, Hanxu Chen^2^, Xiangyi Wu^1^, Yuanjin Zhao^1, 2, 3, 4,^***

^1^ Department of Rheumatology and Immunology, Nanjing Drum Tower Hospital, School of Pharmacy, Nanjing University of Chinese Medicine, Nanjing, 210023 China

^2^ School of Biological Science and Medical Engineering, Southeast University, Nanjing 210096, China

^3^ Shenzhen Research Institute, Southeast University, Shenzhen 518071, China

^4^ Institute of Organoids onChips Translational Research, Henan Academy of Sciences, Zhengzhou 450009, China

Correspondence should be addressed to Yuanjin Zhao; yjzhao@seu.edu.cn


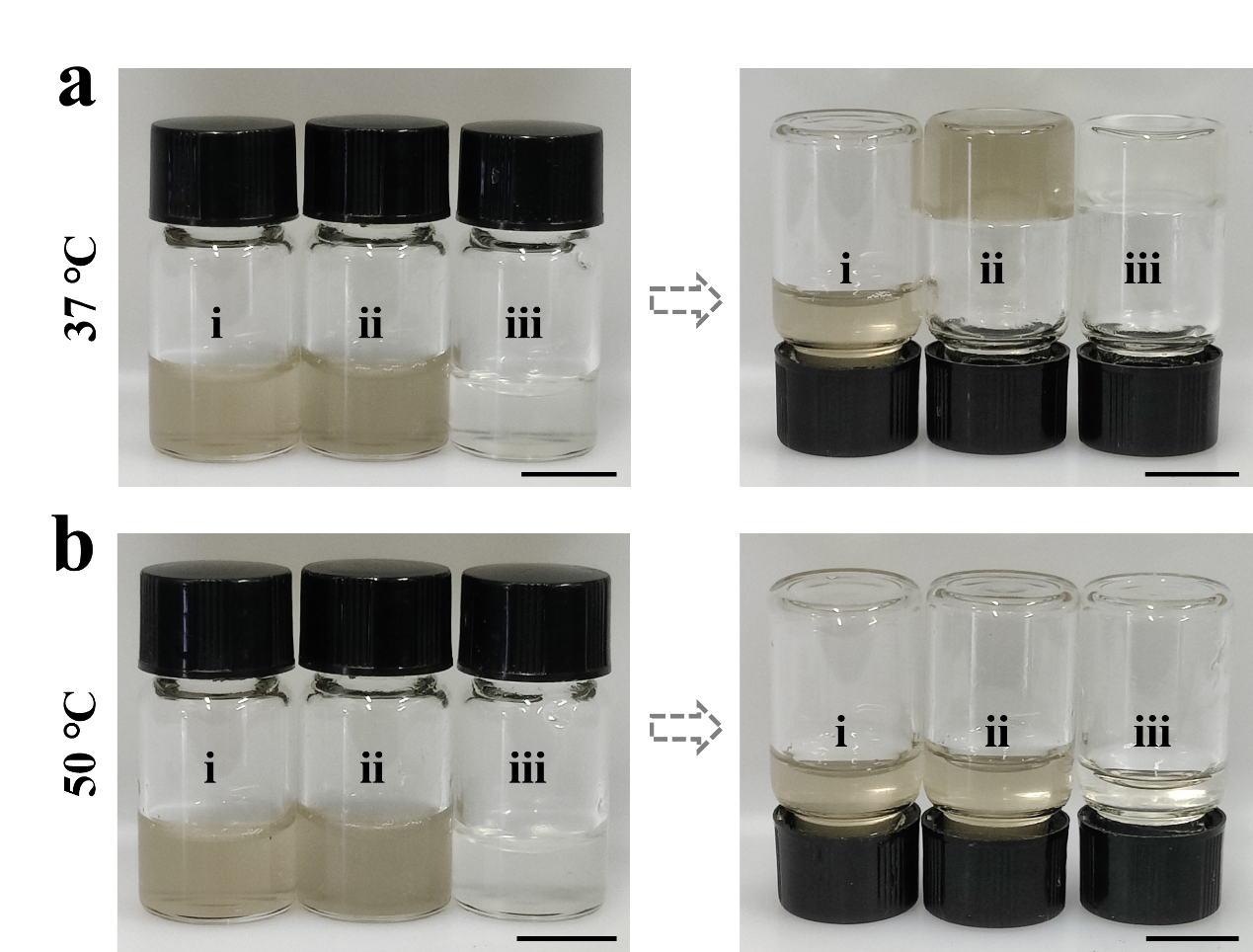


FIGURE S1: (a) Images of (ⅰ) GelMA+BP solution, (ⅱ) GA solution and (ⅲ) GelMA+BP+GA solution in upright and inverted positions at 50 °C. (b) Images of (ⅰ) GelMA+BP solution, (ⅱ) GA solution and (ⅲ) GelMA+BP+GA solution in upright and inverted positions at 37 °C. Scale bar are 1 cm.





FIGURE S2: Size distribution of BMSCs-exo after treatments of 4 ℃, 50 ℃ and UV illumination for 1 min, examined by DLS.


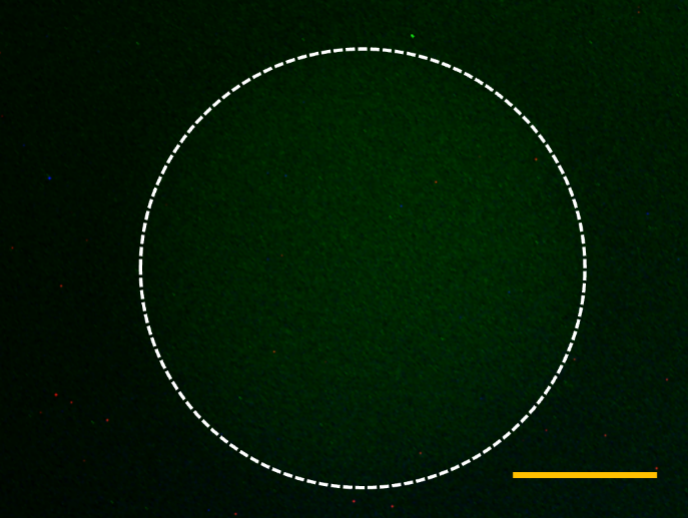


FIGURE S3: Fluorescence image of the BMSCs-exo encapsulated in microparticle. Scale bar is 100 μm.





FIGURE S4: Drug release from GA microparticles in PBS (PH 7.4).


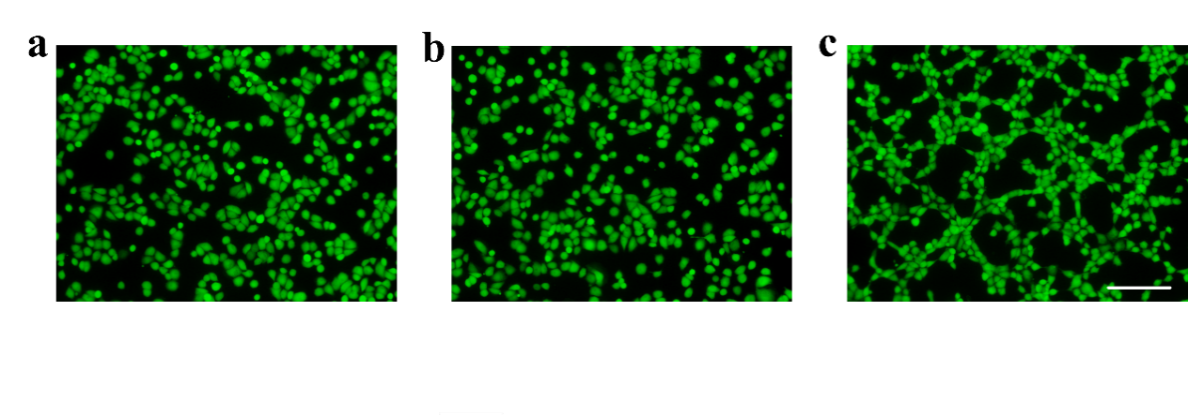


FIGURE S5: (a-c) Fluorescence images of tube formation in (a) the control group (the blank multi-well plate), (b) GA microparticles group (Group Ⅰ) and (c) GA microparticles with BMSCs-exo group (Group Ⅱ). Scale bars are 500 μm in (a-c).


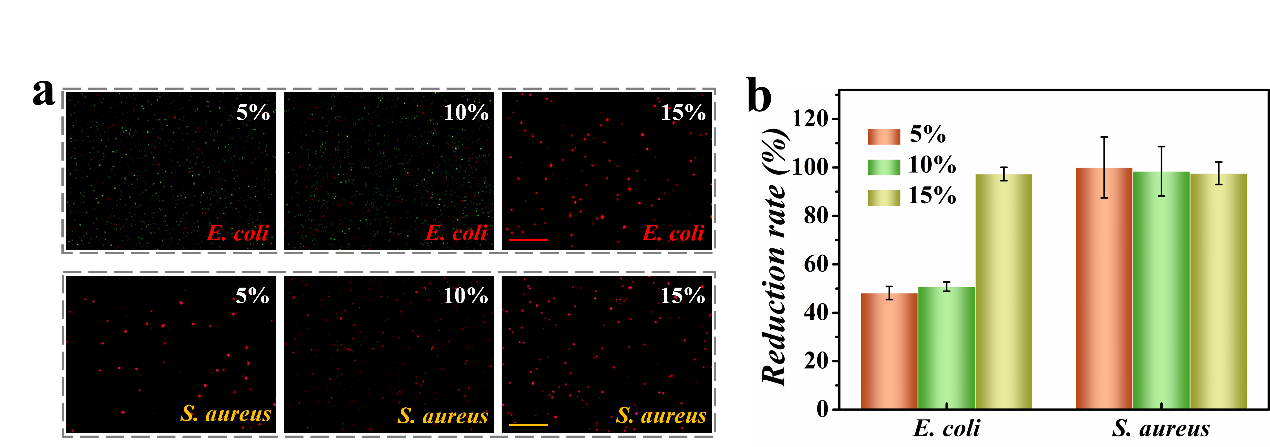


FIGURE S6: (a) Fluorescent images of *E. coli* and *S. aureus* treated with different GA concentrations, stained by SYTO (green) and propidium iodide (PI) (red). (b) The reduction rates of *E. coli* and *S. aureus* in different groups. Scale bars are 50 μm in (a).


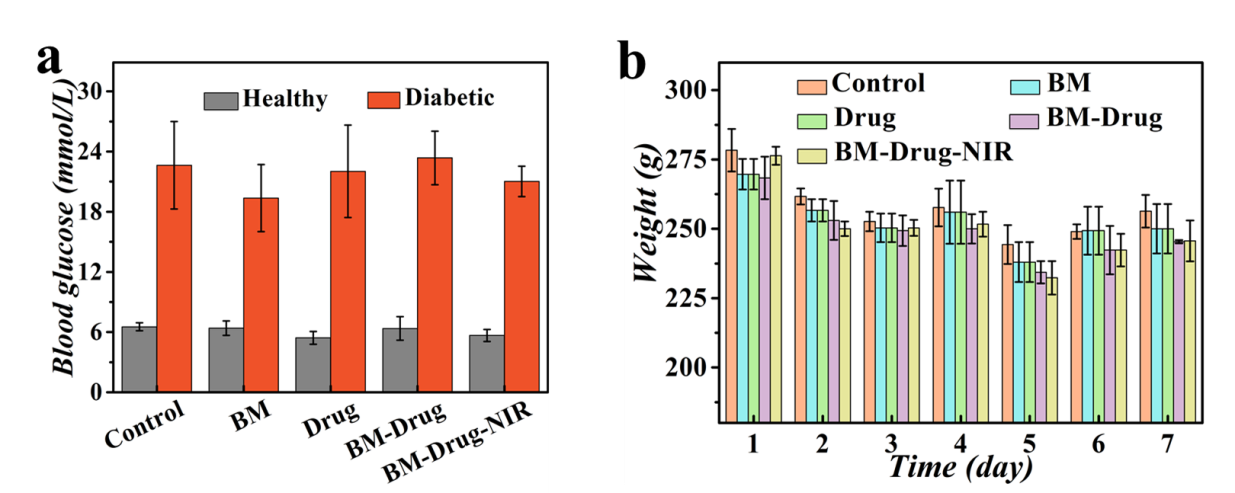


FIGURE S7: (a) Comparison of blood glucose level between healthy SD rats and diabetic SD rats. (b) Changes in body weight in different groups of SD rats after modeling.


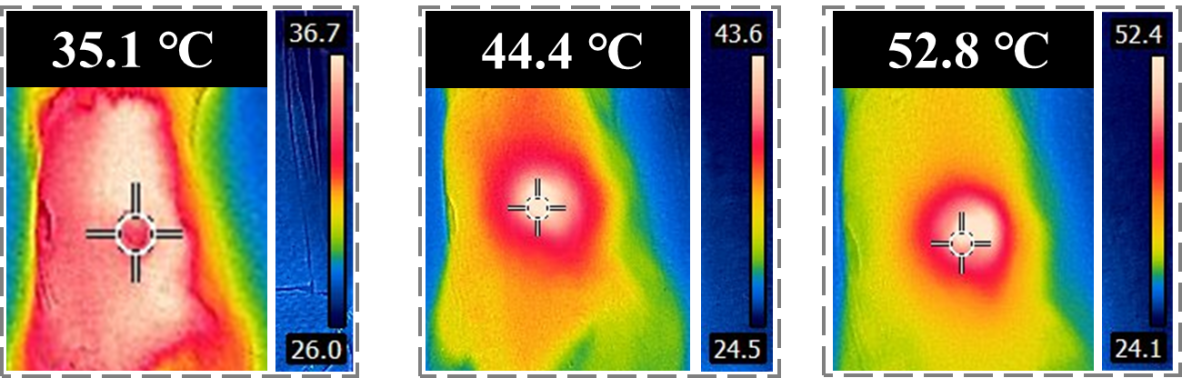


FIGURE S8: Thermal images of wound treated with GA microparticles with BMSCs-exo during 3 min NIR irradiation. The NIR power was 2.69 W/cm^2^.


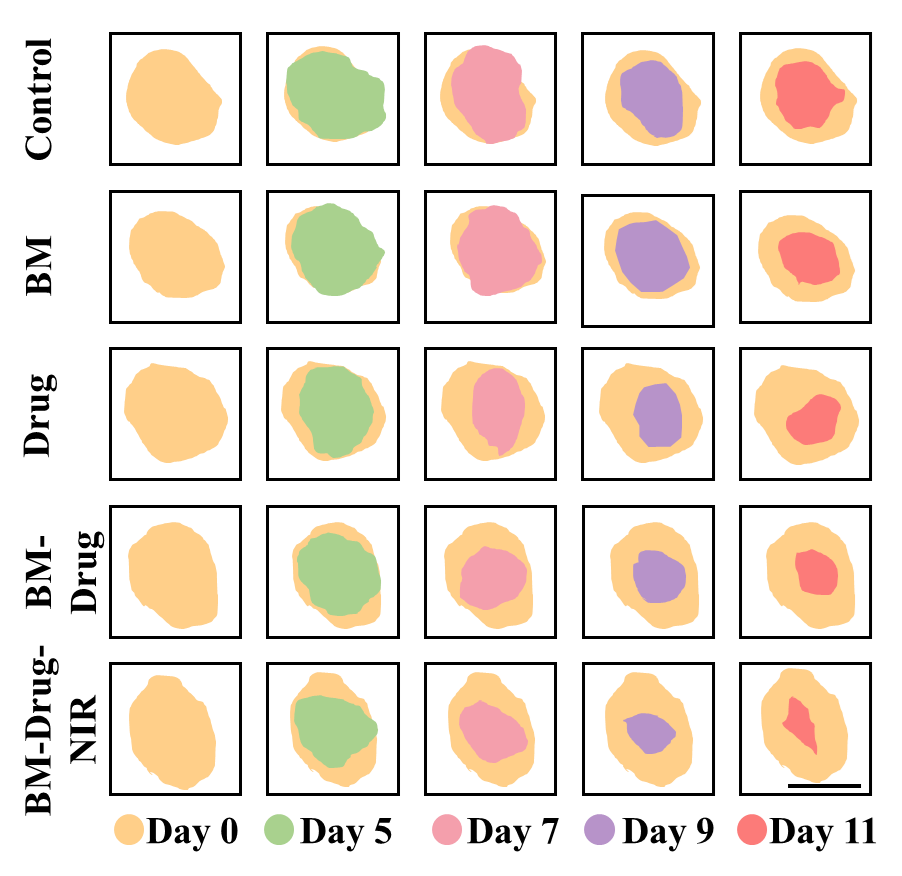


FIGURE S9: Diagram of relative changes in wound area within 11 days. Scale bar is 1 cm.


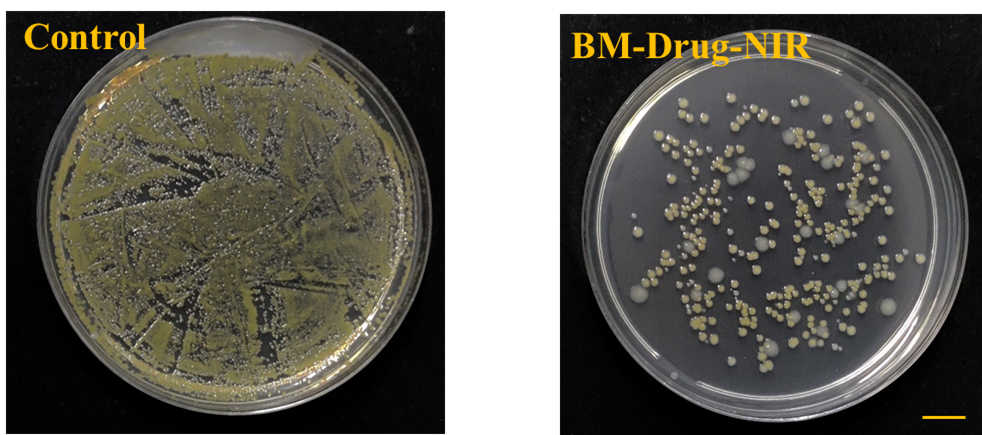


FIGURE S10: *Ex vivo* antibacterial test. Photographs of *S. aureus* colonies in control group and BM-Drug-NIR group on day 2. Scale bar is 1 cm.
